# Transcriptomic analysis of graft liver provides insight into the immune response of rat liver transplantation

**DOI:** 10.3389/fimmu.2022.947437

**Published:** 2022-11-03

**Authors:** Wanyue Cao, Jing Lu, Shanbao Li, Fangbin Song, Junming Xu

**Affiliations:** Department of Hepatobiliary Surgery and Liver Transplantation, Shanghai General Hospital, Shanghai Jiao Tong University School of Medicine, Shanghai, China

**Keywords:** RNA-seq, liver transplantation, regulatory T cells, immune checkpoint, differentially expressed genes, GO, KEGG pathway

## Abstract

**Background:**

As an “immune-privileged organ”, the liver has higher rates of both spontaneous tolerance and operational tolerance after being transplanted compared with other solid organs. Also, a large number of patients still need to take long-term immunosuppression regimens. Liver transplantation (LT) rejection involves varieties of pathophysiological processes and cell types, and a deeper understanding of LT immune response is urgently needed.

**Methods:**

Homogenic and allogeneic rat LT models were established, and recipient tissue was collected on postoperative day 7. The degree of LT rejection was evaluated by liver pathological changes and liver function. Differentially expressed genes (DEGs) were detected by transcriptome sequencing and confirmed by reverse transcription-polymerase chain reaction. The functional properties of DEGs were characterized by the Gene Ontology (GO), Kyoto Encyclopedia of Genes and Genomes (KEGG), and Reactome pathway analyses. The cells infiltrating the graft and recipient spleen and peripheral blood were evaluated by immunofluorescence and flow cytometry.

**Result:**

A total of 1,465 DEGs were screened, including 1,177 up-regulated genes and 288 down-regulated genes. GO enrichment and KEGG pathway analysis indicated that DEGs were involved in several immunobiological processes, including T cell activation, Th1, Th2 and Th17 cell differentiation, cytokine-cytokine receptor interaction and other immune processes. Reactome results showed that PD-1 signaling was enriched. Further research confirmed that mRNA expression of multiple immune cell markers increased and markers of T cell exhaustion significantly changed. Flow cytometry showed that the proportion of Treg decreased, and that of PD-1^+^CD4^+^ T cells and PD-1^+^CD8^+^ T cells increased in the allogeneic group.

**Conclusion:**

Using an omic approach, we revealed that the development of LT rejection involved multiple immune cells, activation of various immune pathways, and specific alterations of immune checkpoints, which would benefit risk assessment in the clinic and understanding of pathogenesis regarding LT tolerance. Further clinical validations are warranted for our findings.

## Introduction

Liver transplantation (LT) is the most effective way to treat end-stage liver disease (1). In the United Kingdom, 1- and 5-year survival rates were reported to be over 90% and 80%, respectively (2). However, patients with long-term application of immunosuppressive (IS) drugs also face the risks of serious adverse events, accounting for 58% of deaths after three years in LT recipients (3, 4). Rejection is one of the important reasons leading to the failure of liver transplantation. Understanding the mechanism of LT rejection and inducing immune tolerance can provide more specific strategies and improve postoperative quality of life.

LT immunity is an adaptive immune response involving activation of T and B lymphocytes (5). T cells can be divided into different subgroups according to their function. Regulatory T cells (Tregs), a specialized subset of CD4^+^ T cells expressing the key transcription factor Foxp3, play an important role in operational tolerance after solid organ transplantation (6, 7). Tregs could inhibit activation and proliferation of multiple immune cells through inhibitory costimulatory molecules, such as T cell immunoglobulin domain and mucin domain 3 (*TIM-3*), programmed cell death protein 1 (*PD-1*), and T cell immunoreceptor with Ig and ITIM domains (*TIGIT*) (8–11). Studies have also shown that costimulatory pathways blockade could limit the activation of T cells reversely (12), which may alter the immune response against allograft and attenuate rejection, prolonging graft survival time (13). Currently, the potential for adoptive cell therapy with Tregs to promote transplant tolerance is being actively explored (14). Although most results of these trials are optimistic, many experimental and clinical unanswered questions are slowing the progression of this new therapeutic alternative (15). In addition, cytokines with pro-inflammatory and regulatory properties are also considered as potential therapeutic targets for inhibiting or enhancing the immune response of recipients (16–18). These results suggest that the content of cytokines, proliferation, and differentiation of T cell subsets, and inhibition of costimulatory molecules are essential for the formation of immune tolerance.

To date, several reports have focused on the effects of different factors on gene expression patterns in regenerating rat livers (19–21), post-transplant tumor recurrence (22), before and after human liver perfusion (23–25), and even the microenvironment of steatotic liver graft (26) using the next-generation RNA sequencing (RNA-seq) or at the single‐cell level. However, to our knowledge, our research is the first to reveal differential expression profiles between immune tolerance and rejection models of rat liver transplantation by RNA-seq, complemented with additional validation using quantitative real-time PCR, immunofluorescence, and flow cytometry. We found that LT rejection involves the participation of various immune cells, the activation of immune process, and the changes of multiple immune checkpoints. These results provide a strong theoretical basis for the potential clinical risks, related immune process, and pathogenesis of LT, and promote us to design rational drugs for the treatment of liver dysfunction caused by LT rejection.

## Materials and methods

### Animals

Kamada’s two-cuff method was used to establish a rat LT model (27). Lewis and Brown Norway (BN) rats, each weighing 210-240 g, were utilized as liver donors and recipients to establish allogeneic rejection models. Lewis-rats were used to construct homogenic tolerance models. All operations were carried out in accordance with the guidelines of the Ethics Committee of Shanghai General Hospital, Affiliated Hospital of Shanghai Jiao Tong University School of Medicine (Ethical code: 2019SQ147).

### Histopathological examination and blood sample testing

Liver tissue was collected from the recipient rats for histopathological examination. The rejection activity index (RAI) was independently determined by three pathologists using the Banff Schema (International Panel, 1997) (28). Serum samples were obtained from the recipients for evaluation of liver function on postoperative day (POD) 7. The levels of alanine aminotransferase (ALT), aspartate aminotransferase (AST), γ-glutamyl transpeptidase (GGT, γ-GT), and total bilirubin (TBIL) were examined with an automated chemical analyzer (Hitachi 7600-10; Hitachi High-Technologies, Japan). The ELISA kits of necrosis factor-β (TNF-β), interferon-γ (IFN-γ), and interleukin-2 (IL-2), IL-4 were purchased from MULTISCIENCE (Shanghai, China).

### RNA isolation, library construction and sequencing

Liver tissues of recipient rats were collected on POD 7. Total RNA was isolated from graft liver tissue using TRIzol reagent (ThermoFisher, Waltham, MA, USA) according to the manufacturer’s guideline, and the purity and concentration of RNA were detected by agarose electrophoresis or a standard Agilent 2100 Bioanalyzer (Agilent Technology, Santa Clara, CA, USA). Briefly, mRNA was captured by magnetic oligo(dT) beads and fragmented, and the first-strand cDNA was generated using random hexamers. After the library was constructed, PCR amplification was used to enrich fragments, and 450bp was optimal size. Then, the total concentration and effective concentration of the library were detected using an Agilent 2100 Bioanalyzer. The standardized cDNA libraries were sequenced on an Illumina HiSeq2500 sequencer by the way of paired-end. The data were stored in the form of FASTQ. The raw sequencing data were filtered and evaluated for quality. The clean reads were mapped to the reference genome (Rattus_norvegicus.Rnor_6.0.dna.toplevel.fa) using BWT algorithm of HISAT2 (29). After the statistical analysis, the software HTSeq-count (30) was used to screen the differentially expressed genes (DEGs) by the following criteria: fold change >2 or < −2, false discovery rate (FDR) < 0.05.

### Bioinformatic analysis

The Gene Ontology (GO) (31, 32) is a free available public resource that describes the role of genes in biological systems. The GO terms were comprised of the following three divisions: biological process (BP), cellular component (CC) and molecular function (MF). Adj. P<0.05 was regarded as statistically significant (33).

Kyoto Encyclopedia of Genes and Genomes (KEGG) is an integrated database resource, which aims to link genomic information with higher order functional information by computerizing current knowledge on cellular processes and by standardizing gene annotations (34).

The Reactome Knowledgebase systematically links proteins to their molecular functions, providing a resource that serves both as an archive of biological processes and as a tool for discovering unexpected functional relationships (35).

The GO annotation, KEGG pathways, and Reactome pathways were used to visualization and pathway enrichment analysis of DEGs, with a corrected P-value < 0.05 defined as significantly enriched.

### Quantitative real-time polymerase chain reaction

RNA was extracted as previously described. The extracted RNA was analyzed by Nano Drop 2000 (Thermo Fisher Scientific, Waltham, MA, USA) to ensure RNA quality and purity. After quantified, RNA was reverse transcribed into cDNA using PrimeScript™ RT Master Mix (Perfect Real Time; Takara). RT‐PCR was performed using SYBR^®^ Premix Ex Taq™ (Tli RNaseH Plus; Takara, Japan) in a Light Cycler Real‐time PCR System (Roche) (36). The corresponding primer sequences are provided in **Supplement Table 1**.

### Immunofluorescence assay

Paraffin sections were de-paraffinized, rehydrated and antigen retrieval according to standard protocols. Then, slices were incubated with anti-CD3 (1:200, Abcam, ab16669), anti-CD4 (1:200, Abcam, ab237722), anti-CD8 (1:200, Abcam, ab33786), and anti-Foxp3 (1:100, Abcam, ab215206). Fluorophore-conjugated secondary antibodies were incubated for one hour at room temperature (1:200, Abcam). The slides were imaged with fluorescence microscopes (Leica, Barcelona, Spain).

### Flow cytometry

Spleens of recipient rats were collected, grounded, and filtered to obtain cell suspension, which was centrifuged at 1600 rpm at 4°C for 5 mins. The precipitation was resuspended by pre-cooled phosphate buffer solution. Double volume of lymphocyte separation solution purchased from Sigma-Aldrich was added and centrifuged at 2000 RPM for 25 min to obtain cell suspension. CD4^+^ T cells and CD8^+^ T cells were isolated by LS column (Miltenyi Biotec, Germany, #130-042-401) with the method of antibiotic microbeads (Miltenyi Biotec, Germany, # 130-090-319, # 130-090-318) as previously described (37, 38). Sorted cells were incubated directly with diluted fluorochrome-conjugated monoclonal antibodies as shown below: anti-CD4 (Invitrogen, #11-0040-82, FITC), anti-CD8a (Invitrogen, #11-0084-82, FITC), anti-PD-1 (Proteintech, #65211, Coralite647) and anti-Foxp3 (eBioscience, #12-5773-82, PE).

### Statistical analysis

Statistical analyses were performed by GraphPad Prism V7.0. Results are shown as representative images or as mean ± standard deviation (SD) of at least three independent experiments. Differences between LT tolerance and rejection groups were estimated by the Student’s *t*-test. For all tests, statistical significance was considered at a *P* value < 0.05.

## Results

### The allogeneic LT group has a higher degree of rejection than the homogeneic group

**Figure 1:**
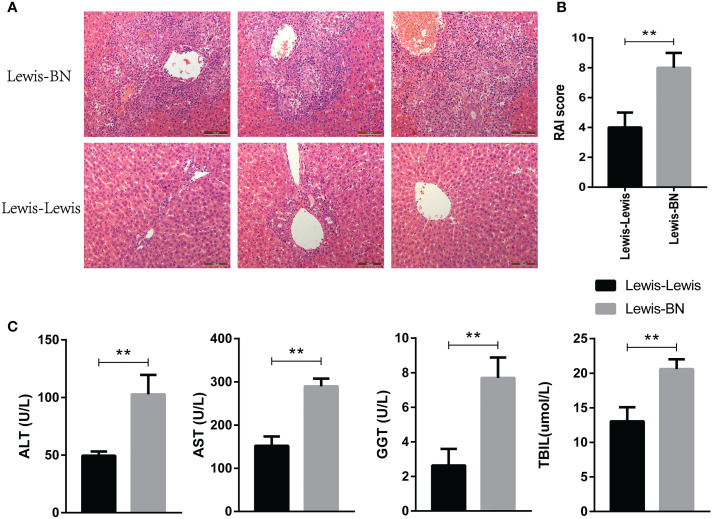
Validation of differences in liver transplantation rejection between Lewis-Lewis group and Lewis-BN group on POD 7. **(A, B)** Hepatic morphologic changes and RAI scores of recipient rats. **(C)** Peripheral blood of each group was taken for analysis and determination of serum aspartate aminotransferase (AST), alanine aminotransferase (ALT), γ-glutamyl transpeptidase (GGT) and total bilirubin (TBIL) by automatic biochemical analyzer (N = 3). *P<0.05, **P < 0.01, ***P < 0.001.

The homogeneic and allogeneic rat LT models were constructed and the rejection degree was evaluated on POD 7. Histopathological changes of liver were graded according to the Banff model (28). As shown in **Figure 1A**, rejection was relentless in the allogeneic LT model, as evidenced by marked infiltration of inflammatory cells into most portal areas, damage of bile duct epithelial cells and increased venous endothelial inflammation. The RAI score was shown in **Figure 1B** (*t=4.899, P=0.008*). In addition, the recipients in the homogeneic LT group shown better liver function indexes compared with allogeneic group (ALT: *t=5.349 P=0.0059;* AST: *t=8.479 P=0.0011;* GGT: *t=5.77 P=0.0045;* TBIL: *t=5.279 P=0.0062*). These results indicated that the allogeneic LT group has a higher degree of rejection than the homogeneic group.

### RNA-seq profiling showed allograft rejection involved significant immune dysregulation

**Figure 2:**
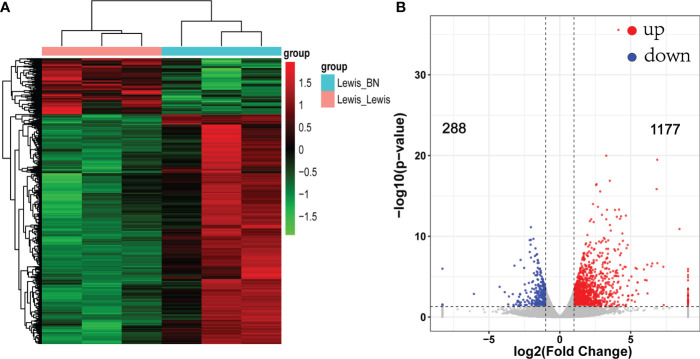
Heat map and volcano map of differentially expressed genes (DEGs). **(A)** Each column represents a sample. Red indicates up-regulated genes; green indicates down-regulated genes. **(B)** Volcano map of DEGs. fold change >2 or < −2, FDR < 0.05.

To reveal the molecular mechanism related to graft rejection, gene expression changes between the two groups were identified by RNA-seq. A total of 845,870,684 reads were obtained. After filtering and quality control, an average of 92.7% reads are available. The allograft models were taken as the experimental group. Using criteria of fold-change > 2 and FDR < 0.05 to define DEGs. Compared with the control group, a total of 1,465 DEGs were identified, of which 1,177 up-regulated genes and 288 down-regulated genes (**Figure 2**). Among them, the top five down-regulated genes were *SLC24A2, Pex5l, Kif28p, Brinp3*, and *Nudt10.* However, the genes related to various immune components significantly upregulated in the allogeneic group with increased transplantation rejection response. The markers of T cell (*CD2,CD3, CD28*), B cell *(CD19,CD79A*), T-cells/NK-cells/T-cell activation and migration (*ICOS, LCK, GZMA/B, CCR6, CCR7*) and cellular infiltrates, such as dendritic cell markers *CD80* and *CD86* were significantly up-regulated. In addition to the markers mentioned above, multiple immune pathways, including the Th1 (*STAT1, TBX21,IFNG, CXCL9/CXCL10/CXCL11*), Th2 (*CCR5, CCL11*) and T cell exhaustion (*LAG3, TIGIT, HAVCR2*) were also up-regulated. The top five genes with the highest expression up-regulation related to immune regulation were *CRTAM, IFNG, TIGIT, IL21, CD3E.* Changes in the expression of chemokine receptor families and markers of immune cells suggest that multiple immune pathways are involved in the activation and participation of liver transplantation rejection.

### DEGs functional annotation and enrichment analysis

**Figure 3:**
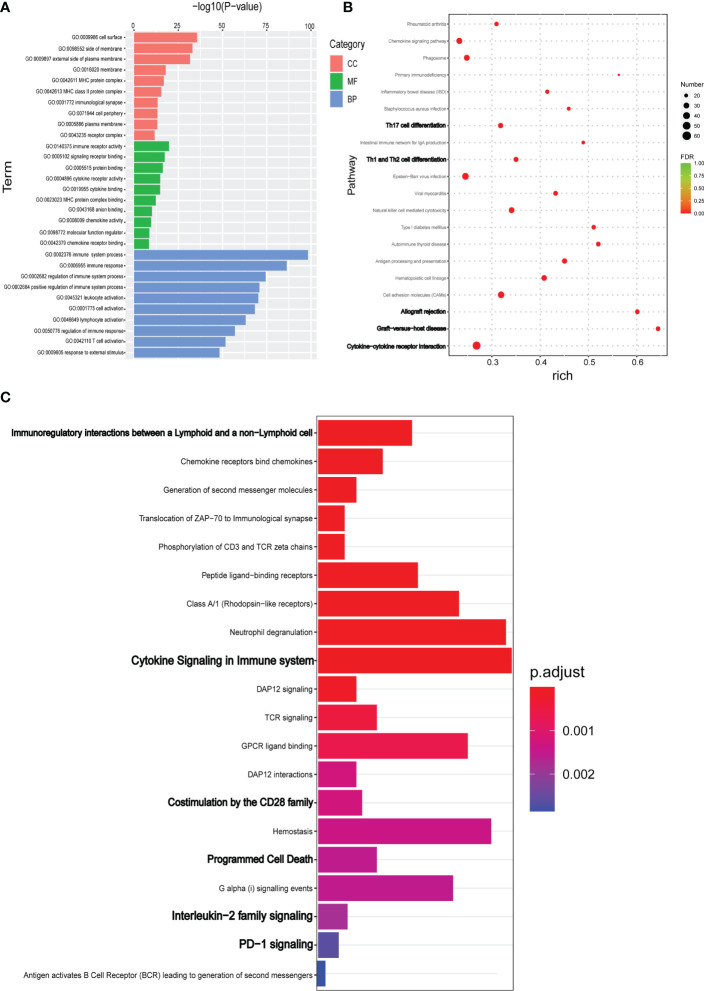
DEGs identified by RNA-seq were evaluated for functional enrichment using multiple gene annotation databases. **(A)** TopGO was used for GO enrichment analysis. GO enrichment analysis results of DEGs were classified according to molecular function (MF), biological process (BP) and cell component (CC). The top 10 GO term items with minimum p-value and most significant enrichment in each GO category were selected. **(B)** KEGG pathway enrichment analysis of DEGs. According to KEGG enrichment results, the top 20 KEGG pathways with the smallest FDR value, namely the most significant enrichment, were selected. **(C)** For the results of Reactome enrichment analysis of DEGs, the top 20 Reactome results with minimum p-value were selected.

DEGs were annotated using the GO database to examine the biological functions and pathways. As shown in the **Figure 3A**, DEGs were mainly involved in the biological processes such as immune response, regulation of the immune system process and T cell activation, etc., which were consistent with the above speculation. The most enriched MF terms include immune receptor activity, signaling receptor binding, protein binding, cytokine receptor activity, and chemokine binding. In addition to other cell components such as cell surface, side of membrane, and external side of plasma membrane, MHC and MHC class II protein complexes are also enriched (**Figure 3A**).

KEGG database was used to functionally annotate the observed gene expression changes to identify potential pathways. KEGG pathway analysis indicated that DEGs were enriched in cytokine-to-cytokine receptor interaction, graft-versus-host disease, allograft rejection, cell adhesion molecules, Th1/Th2/Th17 cell differentiation, NK cell–mediated cytotoxicity, and antigen processing and presentation (**Figure 3B**). In addition to the classic KEGG pathway analysis, the Reactome database was also used to further analyze possible pathways involved in DEGs. As shown in the **Figure 3C**, the main enrichment pathway of DEGs were immunoregulatory interactions between a Lymphoid and a non−Lymphoid cell, Chemokine receptors bind chemokines, Cytokine Signaling in immune system, TCR signaling, Programmed Cell Death, and PD−1 signaling (**Figure 3C**).

### Analysis and validation of RNA-seq data

**Figure 4:**
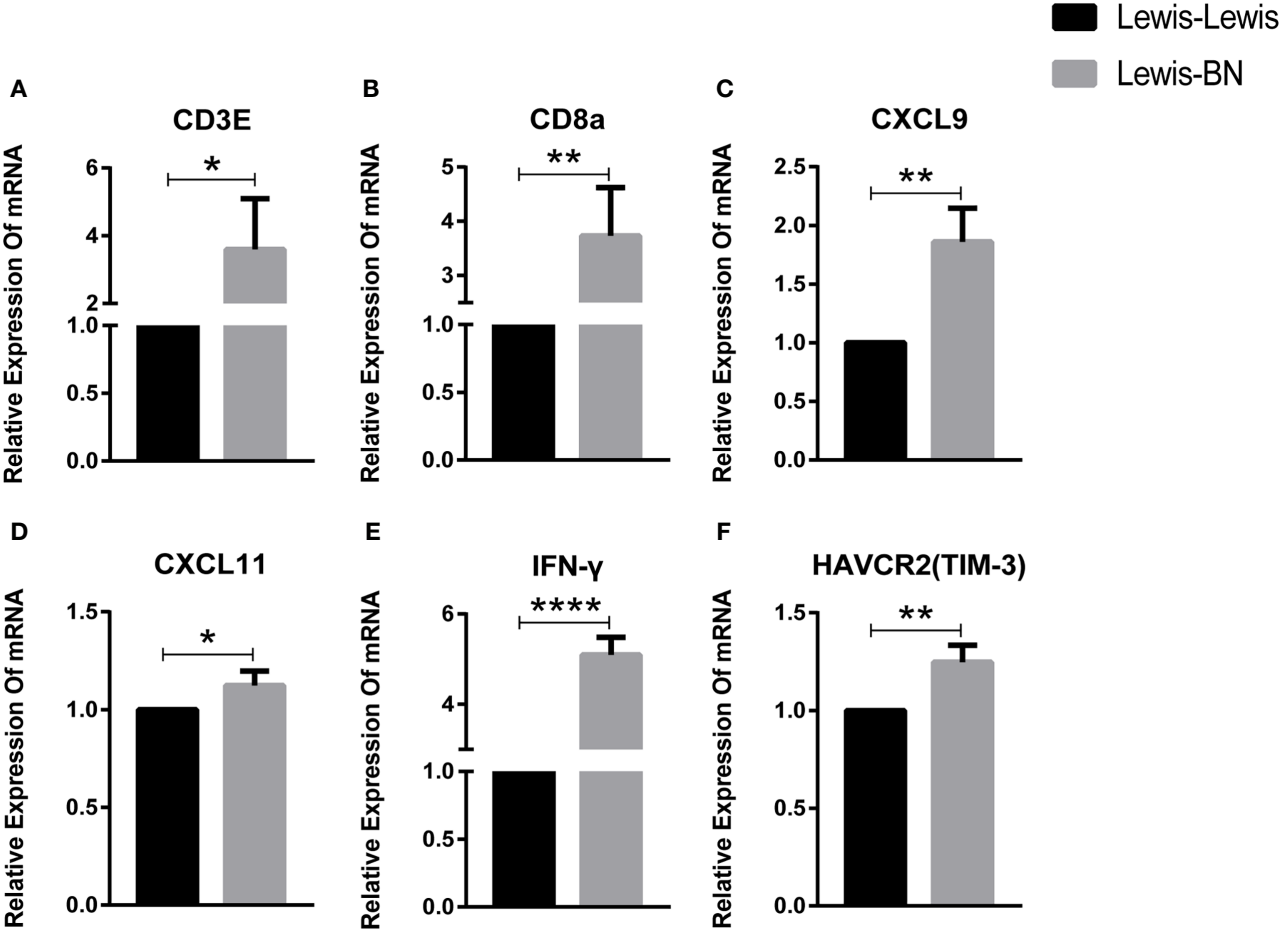
Fold-changes of immune mediators in the liver of homogenic and allogeneic rat liver transplantation recipients were measured by quantitative real-time PCR. *P<0.05, **P < 0.01, ***P < 0.001, ****P < 0.0001.

To further identify key genes involved in the progress of LT rejection, we selected ten genes associated with T cell activation or immunosuppression. The expression of T cell markers, *CD3e* and *CD8a*, increased (*CD3e: P=0.0397; CD8a: P=0.006*), while the expression difference in CD4 was not significant. The main Th1 cytokines, IFN-γ, and Th1 pathway, *CXCL9* and *CXCL11*, were significantly increased (*IFN-γ: P<0.0001; CXCL9: P=0.0065; CXCL11: P=0.442*), while *FOXP3*, the Treg marker and Th17 cytokine, IL-17a, showed no significant changes. In addition, the marker of T cell exhaustion *TIM-3* showed significant differences *(P=0.0077*), but *PDCD1* mRNA did not differ between the two groups (**Figure 4**).

### The cells infiltrating graft liver were increased in the rejection group

**Figure 5:**
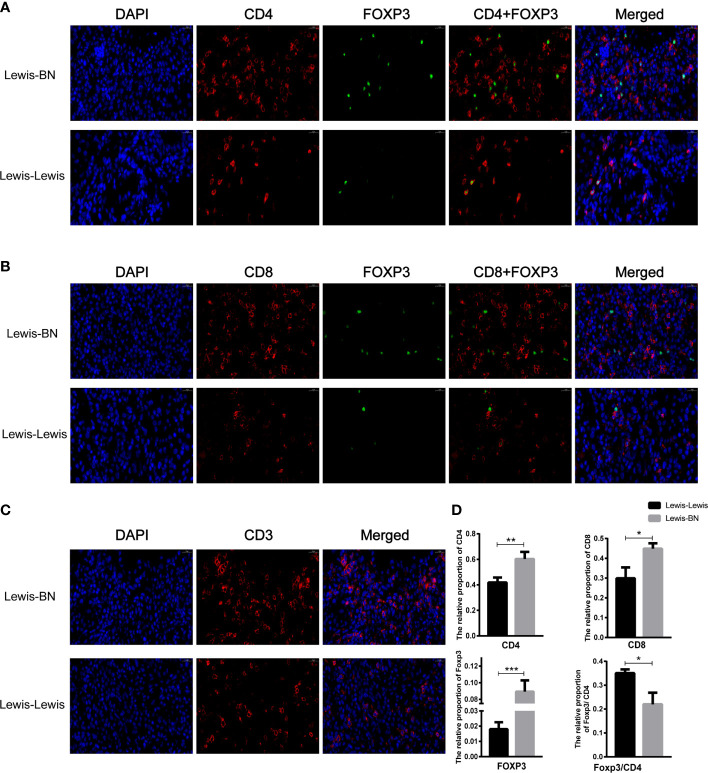
Comparison of the content of cells infiltrating graft between two groups. **(A-D)** The cells infiltrating the graft were examined by immunofluorescence staining. *P<0.05, **P < 0.01, ***P < 0.001.

**Figure 6:**
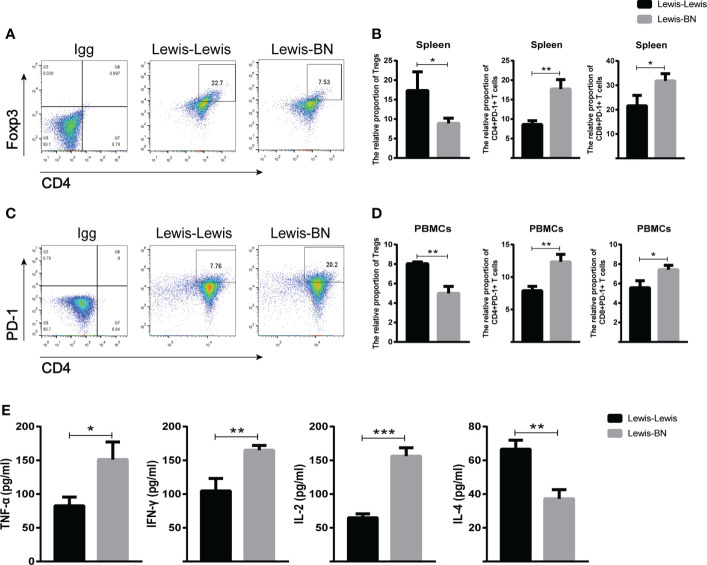
**(A–D)** Spleen and peripheral blood of recipient rats in each group was collected on POD 7. The content of CD4+Foxp3+, CD4+PD-1, CD8+PD-1 T cells were detected by flow cytometry. **(E)** The serum levels of TNF-β, IFN-γ, IL -2 and IL-4 in each group was detected on POD 7. *P<0.05, **P < 0.01, ***P < 0.001.

We further explored the cells infiltrating graft to confirm the reliability of the expression profiles generated by the RNA-Seq and DEGs analysis. Compared with the control group, the content of CD4 and CD8 T cell was up-regulated in the allograft (CD4^+^ T cell*: t=4.641, P=0.097;* CD8^+^ T cell: *t=4.201, P==0.0137*), and the expression of *Foxp3* was also increased (t=8.665, P<0.001). However, the ratio of Foxp3/CD4 showed an opposite trend in allograft (*t=4.433, P=0.0114*) (**Figure 5**). We also investigated the proportion of Foxp3^+^ T cells and the expression of *PD-1* in the recipient spleen and PBMCs. The expression level of *Foxp3* on T cells showed lower expression on CD4^+^ T cells compared with the control group (Spleen: Tregs, *t=2.942, P=0.0423*, PBMCs: Tregs, *t=7.166, P=0.002*). The expression of *PD-1* on CD4^+^ and CD8^+^ T cells was up-regulated with the aggravation of rejection (Spleen: PD-1^+^CD4^+^ T cell, *t=6.106, P=0.036;* PD-1^+^CD8^+^ T cell, *t=3.425, P=0.02676;* PBMCs: PD-1^+^CD4^+^ T cell, *t=5.75, P=0.0045;* PD-1^+^CD8^+^ T cell, *t=3.818, P=0.0188*) (**Figures 6A–D**). The above results were consistent with the previous results of DEGs analysis. The proportion of CD4^+^ T and CD8^+^ T cells in the transplant rejection group was increased, and the expression of immune checkpoint *PD-1* was also up-regulated. However, the proportion of Tregs was higher in the control group.

### Comparison of cytokine content in peripheral blood

Serum cytokines secreted by T cells play an important role in regulating various immune responses, including LT (39). Therefore, we measured the levels of TNF-α, IFN-γ, IL-2, and IL-4 by ELISA kit. The results indicated that TNF-α, IFN-γ and IL-2 increased in allogeneic transplant recipients (TNF-α: *t=4.072, P=0.0152;* IFN-γ: *t=5.31, P=0.006;* IL-2: *t=11.62, P=0.0003*) while IL-4 decreased (*t=6.641, P=0.0027*) (**Figure 6E**). Together, these results further suggest that multiple cytokines secreted by Th1 and Th2 cells are involved in the immune rejection of LT.

## Discussion

Compared with other solid organs, a small portion of stable LT recipients showed sustained graft tolerance after complete withdrawal from IS, known as operational tolerance (40). However, the majority of patients still require a lifetime medication regimen. Therefore, the processes and mechanisms that control the outcome of LT remain to be thoroughly understood.

A major advantage of RNA-seq is its ability to identify potential novel biomarkers (41). The high genomic match between rat and human makes it a powerful model for elucidating the immune mechanisms of LT (42). There have been numbers of reports on liver regeneration pathways, ischemia-reperfusion at different time points, and different perfusion methods of rats and humans (19, 22, 24, 25, 43). Single-cell techniques have also been reported to assess the plasticity and phenotype of immune cells in the microenvironment of liver grafts (26). However, mRNA changes in rats at 7 days after homo/allogeneic LT have not been reported. And this is the first comprehensive transcriptomic analysis of rat graft liver on POD 7. DEGs identified were enriched in multiple immune processes, regulation of immune response, lymphocyte activation, and cell activation. KEGG results showed that Th1, Th2, and Th17 cell differentiation, NK-cell-mediated cytotoxicity, antigen presentation, and other immune mechanisms participated in the progression of transplant rejection. In addition to the involvement of the immune system, Reactome database analysis showed that *PD-1* signaling pathway and cytokine signaling were also involved.

The rejection of allogeneic transplantation is mainly mediated by the recognition of non-self donor alloantigens (44–46), which leads to T cell activation and proliferation. MHC class I molecules present intracellular epitopes to CD8^+^ cytotoxic T cells, while MHC class II molecules are mainly responsible for presenting epitopes from extracellular substances to CD4^+^ helper T cells (47). Activated T cells work through a variety of effector mechanisms, including T cell-mediated direct damage to bile ducts, endothelium, and liver cells, as well as indirect effects through cytokine production and tissue destructive inflammatory cell recruitment (44, 48, 49). These effects explain the histological appearance of typical acute T cell-mediated rejection (48). CD8^+^ T cells could differentiate into cytotoxic T-cells able to exert direct cell damage on the allograft (44), and are also the main effector lymphocytes responsible for mediating tissue damage.

The outcome of liver is determined by the balance of effector and regulatory immune cell activities (50). Tregs are thought to be involved in inducing LT immune tolerance (45, 51–55). At present, several clinical trials on Treg treatment are under way (56), but there are still great challenges regarding the efficacy and safety of Treg treatment before it is truly implemented in routine clinical application. We analyzed the infiltrating cells in the liver and functional T cell subsets in the spleen and peripheral blood and found that the proportion of CD4^+^ T cells and CD8^+^ T cells, as well as the expression of *Foxp3*, were significantly increased in the rejection group. However, Foxp3/CD4 ratio decreased, suggesting that the content of Treg cells increased in the tolerant group. In addition, our data further indicated the important role of Treg cells in inducing immune tolerance in liver transplantation.

Most scholars believe that PD-1 plays a crucial role in inducing and maintaining the tolerance of peripheral transplant (57–59), besides, it is also involved in T cell exhaustion. overexpression of PD-1 on CD8^+^ T cells can induce cancer cells to escape from anti-tumor immune response and promote transplant tolerance (60–62). Similar to PD-1, TIGIT, a novel immune checkpoint, which is mainly expressed on NKs, CD8^+^ T cells, CD4^+^ T cells, and Treg cells (63), is well known for its important role in tumor immunity and autoimmune diseases (64–66). In transplantation immunity, *TIGIT* can regulate the severity of graft-versus-host disease (GVHD) by affecting the function of Treg cells and the number of donor antigen-reactive T cells (67–69). However, our results were not consistent with prevailing thinking. Reactome analysis indicated that DEGs were enriched in the activation of *PD-1* signaling pathway. The expression of *PD-1* on CD4^+^ T and CD8^+^ T cells in spleen and peripheral blood showed an increasing trend. And the mRNA expression of *LAG3, HAVCR2(TIM-3)*, and *TIGIT* were significantly up-regulated in the rejection group, while *PDCD1* was not. Currently, there are few studies on the simple application of immune checkpoint inhibitors (ICIs) in LT patients. Most patients suffer from malignant tumors or liver cancer before LT (70–73). Therefore, accurate conclusions cannot be drawn about the role of immune checkpoints in liver transplantation rejection. The mechanism of immune checkpoints such as *PD-1*, *LAG3*, and *TIM-3* in liver transplantation remains to be further studied.

The activation and differentiation of T cells depend on the selection of costimulatory molecules and the composition of cytokines in the environment (74). On the one hand, cytokines secreted by Th1 cells, such as IFN-γ, TNF-α, and IL-2, can adversely affect the graft by recruiting and activating effector T cells (16, 17, 75). On the other hand, immunomodulatory cytokines secreted by Th2 cells, such as IL-4 and IL-10, can induce tolerance to liver allografts (16, 39, 76). Our results showed that TNF-α, IFN-γ, and IL-2 increased in allograft recipients, while IL-4 decreased. It is consistent with the above conclusion. Besides, the content of cytokine can also significantly affect the expression of *PD-1*/*PD-L1 (* 77, 78). *PD-1* expression was found to be elevated in spleen and peripheral blood, we speculated that the content of cytokine may also play a part in it. To sum up, the most important thing is to regulate the balance of various cytokines and use their advantages and disadvantages to achieve the maintenance of homeostasis.

There are some limitations to this study. First of all, the sample size is small. Although the current results show a significant difference in gene expression between the homogeneic group and the allogeneic rejection group, future studies still need a larger cohort and other strains of rats to evaluate its accuracy. Secondly, our study only proposed the hypothesis of possible immune-based treatments for LT, which needs to be confirmed in future proof-of-concept studies and clinical trials.

In summary, the current genomic and cellular profiling study of rat liver transplantation firstly provides a comprehensive molecular fingerprint of the immune alterations between the allogeneic group and the homogeneic group. Our results reveal a broad spectrum of immune system regulation including significant changes in T lymphocytes and a variety of cytokines, as well as maladjustment of immune checkpoints in liver transplantation rejection, providing a novel and promising insight into the understanding and induction of self-tolerance in LT.

## Data availability statement

The data presented in the study are deposited in the GEO repository, accession number GSE210164.

## Ethics statement

The animal study was reviewed and approved by The Ethics Committee of Shanghai General Hospital, Affiliated Hospital of Shanghai Jiao Tong University School of Medicine. Ethical code: 2019SQ147.

## Author contributions

CW participated in research-design; LJ and LS participated in the performance of the research; SF was responsible for the design. XJ provided guidance on the research. All authors contributed to the article and approved the submitted version.

## Funding

This study was funded by the National Natural Science Foundation of China (grant number 81670595 and 81970568).

## Conflict of interest

The authors declare that the research was conducted in the absence of any commercial or financial relationships that could be construed as a potential conflict of interest.

## Publisher’s note

All claims expressed in this article are solely those of the authors and do not necessarily represent those of their affiliated organizations, or those of the publisher, the editors and the reviewers. Any product that may be evaluated in this article, or claim that may be made by its manufacturer, is not guaranteed or endorsed by the publisher.

## Abbreviations

LT, Liver transplantation; IS, Immunosuppressive drugs; Th, Helper T cells; Tregs, Regulatory T cells; TIM-3, T cell immunoglobulin domain and mucin domain 3; PD-1, Programmed cell death protein 1; TIGIT, T cell immunoreceptor with Ig and ITIM domains; RNA-seq, RNA sequencing; TNF-β, Tumor necrosis factor-β; IFN-γ, Interferon-γ; IL-2, Interleukin-2; PBMCs, Peripheral blood Mononuclear Cells; RAI, Rejection activity index; POD, Postoperative day; ALT, Alanine aminotransferase; AST, Aspartate aminotransferase; GGT, γ-GT, γ-glutamyl transpeptidase; TBIL, Total bilirubin; DEGs, Differentially expressed genes; FDR, False discovery rate; (GO, Gene Ontology; BP, Biological processes; MF, Molecular factor; CC, Cell components; KEGG, Kyoto Encyclopedia of Genes and Genomes; GVHD, graft-versus-host disease; ICIs, Immune checkpoint inhibitors.
