# Supplementary material for: Transcriptomic analysis of graft liver provides insight into the immune response of rat liver transplantation
Source: Front Immunol. 2022 Nov 3;13:947437. doi: 10.3389/fimmu.2022.947437 (PMC9680555; doi:10.3389/fimmu.2022.947437)
Supplement: Supplementary file 1 [file Table_1.docx]

**Table S1**

| **Gene** | **Forward (5′-3′)** | **Reverse (5′-3′)** |
| --- | --- | --- |
| **CD3e** | **TTCAAGATAGAAGTGGTTGAATATG** | **CACCTCCTTCGCCAGCTCC** |
| **CD4** | **TGTGTCAGGTGCCGGCACCAACAG** | **GTGGGGCCCAGGCCTCATATG** |
| **CD8a** | **AGGGAATGGGATTGGGCTTCGC** | **CTCTGAAGGTCTGGGCTTGAC** |
| **CXCL 9** | **TGTGGAGTTCGAGGAACCCT** | **ACCCTTGCTGAATCTGGGTC** |
| **CXCL11** | **TTACCCGAGTAACGGCTGTG** | **TGAGGGCCCATCACATGTTC** |
| **IFN-γ** | **CGCTACACACTGCATCTTGG** | **TCCTTTTGCCAGTTCCTCCA** |
| **Foxp3** | **AGTGCCTGTGTCCTCAATGGTC-3** | **AGGGCCAGCATAGGTGCAAG** |
| **IL-17a** | **ACTACCTCAACCGTTCCACG** | **TTCCCTCCGCATTGACACAG** |
| **PDCD1** | **GCGTCTGTGGGTTCTGTGTCG** | **CCAAGGGTGACTTTAGGTGCTG** |
| **TIM-3** | **GCTACGTCAACAGCCAGCAG** | **CCAATGAGGTTGCCAAGTGA** |
| **GAPDH** | **CATCAACGACCCCTTCATTGAC** | **ACTCCACGACATACTCAGCACC** |

Primer sequences used for quantitative real-time PCR analyses.
